# Defining the Exposome Using Popular Education and Concept Mapping With Communities in Atlanta, Georgia

**DOI:** 10.3389/fpubh.2022.842539

**Published:** 2022-04-12

**Authors:** Erin Lebow-Skelley, Lynne Young, Yomi Noibi, Karla Blaginin, Margaret Hooker, Dana Williamson, Martha Scott Tomlinson, Michelle C. Kegler, Melanie A. Pearson

**Affiliations:** ^1^HERCULES Exposome Research Center, Rollins School of Public Health, Emory University, Atlanta, GA, United States; ^2^HERCULES Stakeholder Advisory Board, Atlanta, GA, United States; ^3^Pathways to Sustainability, Duluth, GA, United States; ^4^Environmental Community Action (ECO-Action), Atlanta, GA, United States; ^5^Dichos de la Casa, Norcross, GA, United States; ^6^Department of Behavioral Sciences and Health Education, Rollins School of Public Health, Emory University, Atlanta, GA, United States; ^7^Emory Prevention Research Center, Rollins School of Public Health, Emory University, Atlanta, GA, United States

**Keywords:** exposome, environmental health, community, community engagement, concept mapping, transdisciplinary research, social determinants of health, popular education

## Abstract

**Introduction:**

The exposome concept provides a framework to better incorporate the environment into the study of health and disease and has been defined by academics to encompass all lifetime exposures including toxicants, diet, and lifestyle choices. However, initial applications of the exposome concept have been less apt at measuring social determinants of health, focusing primarily on conventional environmental exposures and lifestyle choices that do not reflect the complex lived experience of many communities. To bring community voice into the exposome concept, the HERCULES Exposome Research Center and its Stakeholder Advisory Board co-developed the Exposome Roadshow. We present and discuss the resulting community-exposome definition to inform and improve exposome research.

**Materials and Methods:**

Four communities from distinct areas across metro-Atlanta participated in separate 2-day Exposome Roadshow workshops with concept mapping. Aligned with a popular education approach in which community knowledge is used to work collectively for change, concept mapping provided a systematic method to collect and visualize community members' knowledge and create a shared understanding to take action. Community members brainstormed, sorted, and rated their responses to the prompt: “What in your environment is affecting your and your community's health?” Responses were analyzed and visually depicted by concept maps consisting of separate but interrelated clusters of ideas. Community members discussed and validated the maps, selecting a final map illustrating their community's exposome.

**Results:**

A total of 118 community members completed concept mapping. On average communities identified 7 clusters to define their exposome. The resulting concept maps offer a community definition of the exposome. Five major themes arose across all four communities: conventional environmental concerns, built environment, social relationships, crime and safety, and individual health and behaviors.

**Discussion:**

The resulting community-exposome definition demonstrates the importance of expanding the scope of exposures beyond traditional environmental influences to include the lived experience of individuals and communities. While newer exposome definitions align more closely with this community definition, traditional exposome methods do not routinely include these factors. To truly capture the totality of lifetime exposures and improve human health, researchers should incorporate community perspectives into exposome research.

## Introduction

The definition of the exposome has been modified, expanded, enhanced, and criticized by academic scholars since 2005, when Wild (1) first introduced the concept. While each evolution of the definition provides additional guidance to scientists studying the link between the environment and disease, the perspective of communities facing environmental challenges has yet to be integrated into the exposome definition. To address this critical omission and improve the validity of the exposome concept, the Emory University HERCULES Exposome Research Center, funded by the National Institutes of Environmental Health Sciences (NIEHS), partnered with Atlanta-based communities to document their exposome-relevant perspectives and lived experiences.

Wild [(1) p. 1848] originally conceptualized the exposome as encompassing all “life-course environmental exposures (including lifestyle factors), from the prenatal period onwards.” Commendably, this first conceptualization included a broad definition of the environment, beyond traditional toxicants with the inclusion of “lifestyle factors.” Yet, Wild's call for methodologic developments in exposure assessment and refined questionnaire-based approaches limited lifestyle factors to the individual without consideration of the social determinants of health. In 2010, Rappaport and Smith (2) enhanced the exposome definition by emphasizing the internal chemical environment relative to external toxicants. Importantly, these scholars depicted the external environment to include psychosocial factors, a necessary first step in broadening the exposome beyond the individual. Soon after, Wild expanded his definition of the external environment to include the specific external and general external environments. This expansion encompassed both the traditional environmental risk factors and the more generalized environmental factors inclusive of social determinants of health. Importantly, Wild (3) called for interdisciplinary teams, including social scientists, to measure the exposome, while Miller and Jones (4) refined the definition to be quantifiable.

Despite the recognized need for social scientists' involvement in the exposome, many of the methodologies proposed and applied to exposome science continue to be limited to the fields of toxicology, epidemiology, laboratory science, and data science (5). To address these limitations, Juarez and colleagues (6) proposed the public health exposome to capture the complexity of the relationships between the environment and health disparities at a population level. The public health exposome places the exposome paradigm within a social-ecological framework, from which Juarez and colleagues (6) created a data repository and bioinformatics infrastructure that integrates GIS for visualization and analytics. Similarly, the socio-exposome framework describes three levels from which to analyze environmental exposure data: individual, local, and global (7). Notably, both the public health exposome and socio-exposome frameworks emphasize the need for community engagement in exposome science (6, 7). Robinson et al. (8) operationalize aspects of both the public health exposome and the socio-exposome in their approach to the urban exposome, which focuses on exposures specific to the urban environment.

This brief summary and timeline of the exposome definition reflects the evolution from recognizing that “every individual has a personal exposome” to understanding that “many parts of the exposome, including exposure levels and correlations, are shared between groups due to shared determinants.” [(8) p. 077005-2]. Yet, many applications of the exposome continue to emphasize molecular level differences and the identification of exposures, ignoring the social context that contributes to the exposures (7). The community voice, which could help to contextualize these exposures, continues to be non-existent in the definitions and applications of the exposome, even among scholars who emphasize community engagement in exposome science (6, 7). This gap was identified by the HERCULES Stakeholder Advisory Board (SAB), which is made up of representatives from local communities and non-profits, government agencies, and academic institutions. To address this gap, the SAB proposed the Exposome Roadshow (the Roadshow), a community-engaged approach to bring community voice to the exposome concept while supporting communities to utilize the exposome concept in their efforts to address local concerns. The Roadshow, the first phase in a larger community grant program, sought to provide education to communities about the exposome, learn from communities about their exposome-related knowledge and concerns (i.e., how they define their exposome), and work together to address those concerns. Importantly, while the Roadshow informed exposome science, it also purposely benefited the community by providing them a systematic approach to conceptualize their issues and a pathway to address these issues. As recommended by the HERCULES SAB, the Roadshow follows a popular education approach, “a philosophy and methodology that seeks to bring about more just and equitable social, political, and economic relations by creating settings in which people who have historically lacked power can discover and expand their knowledge and use it to eliminate societal inequities” [(9) p. 38]. Popular education draws on personal and collective knowledge and experience as the foundation for shared learning, understanding, and action (10) and, as such, we sought to learn the community's exposome definition before sharing our own. To do this, we used concept mapping, a methodology aligned with popular education philosophy. We then compare the academic definitions of the exposome to the community definition, assess whether or not exposome science is being implemented to capture the community definition, and suggest approaches for incorporating community perspectives into exposome science.

## Materials and Methods

The Roadshow is part of the Clarence “Shaheed” DuBois Exposome Roadshow and Community Grant Program, a four-phase program consisting of the Roadshow, a Planning Grant, Action Grant, and Sustainability Grant. The program details were developed by a workgroup consisting of HERCULES SAB members and staff (the co-authors). The full program [described elsewhere (11)] is aligned with popular education philosophy, drawing on community knowledge and experience to work collectively for change (10). Starting with the Roadshow, concept mapping creates a shared understanding of the community members' collective exposome, which they transform into collective action via the grant program. Community members participated in concept mapping during two Roadshow sessions. The concept mapping steps, as defined by Trochim (12), are outlined in Table 1.

**Table 1:** Concept mapping steps [adapted from Trochim (12)].

**Concept mapping steps**	**Step details**	**Roadshow process**
**Before session one:**		
Step 1 Preparation	Selecting Participants Developing the Focus - Focus for Brainstorming - Focus for Rating	- Develop prompt and rating statement (co-developed with HERCULES SAB) - Program application and review - Identify representative participants (in consultation with community liaison)
**During session one:**		
Step 2 Generation of Statements	Brainstorming	- Exposome analogy presentation (prompt background)
Step 3 Structuring of statements	Sorting Statements Rating Statements	- Brainstorming - Exposome education - Sorting and Rating
**Between sessions:**		
Step 4 Representation of Statements	Creation of Maps	- HERCULES staff use Concept Systems to create different maps - Consultation with community liaison
**During and after session two:**		
Step 5 Interpretation of Maps	Statement List Cluster List Naming the Clusters Point Map Cluster Map Cluster Rating Map	Interpretation of maps: - Presentation of cluster list and cluster rating map to community - Review and validation of cluster list by community - Naming the clusters - Selection of final Concept Map
Step 6 Utilization of maps	Prioritization Action Planning	Utilization of maps: - Select a priority cluster - Begin developing an action plan - Community enters Roadshow Community Grant Program - Identifying community definition of the exposome

While the concept mapping software that we used, Concept System® Global MAX^TM^ (Concept Systems, Inc., Ithaca, USA, Copyright 2004–2020; all rights reserved), provides an online platform, we conducted the concept mapping process in person over the two workshop sessions so as not to limit participation based on technology literacy or access. The steps conducted in each session, as well as before and in between, are presented in Table 1. The Emory Institutional Review Board (IRB) reviewed the Roadshow protocol and determined that it did not meet the definition of research with human subjects.

Four Atlanta-area communities participated in concept mapping as part of the Roadshow between September 2017 and November 2019. The first community to participate volunteered to pilot test the Roadshow. The other three participating communities applied through a formal application process for the larger grant program, which prioritizes environmental justice communities in the 10-county metro Atlanta area (13) that face longstanding disinvestment and multiple compounding issues. As part of the application process communities identified liaisons committed to leading the effort. Participating communities were selected by a review committee composed of SAB members (Step 1: Participant Selection).

Preparation for the Roadshow also included the development of an exposome analogy to provide community members with background on the exposome concept, a Brainstorming prompt/focus to learn the community's definition of the exposome, and a Rating prompt/focus to learn which health influences community members prioritized. Both prompts changed slightly over time (Table 2). Community liaisons helped finalize the prompts and attempted to recruit 25–35 people representative of the larger community to attend the workshops. Participants were given a $25 gift card for each session they attended.

**Table 2:** Brainstorming and rating details for each community.

	**Community A and Community B**	**Community C and Community D**
Brainstorming Prompt^a^	“What influences and has influenced your health?”	“What in your environment is affecting your and your community's health?”
Additional brainstorming guidance^a^	Consider what has influenced your health across your lifespan in all areas in which you spend time	When you think about your environment, please consider the surroundings or conditions in which you live, you are not limited to the natural/geographical environment.
Rating prompt^b^	“On a scale of 1–5, how important is each item to your health?”	“On a scale of 1–10, how important is each item to your health, your family's health, or your community's health?”
Rating scale^b^	1–5	1–10

a *After reflecting on the outcomes from the two initial communities and on the second purpose of the Roadshow, which is to prepare the community to take action on a community priority, we chose to revise the prompt and guidance to help focus on their current community*.

b *Based on our experience with the first two communities, we revised the rating question to provide clarity and to encourage a broader range of ratings*.

During the first workshop session, participants individually brainstormed responses to the prompt (Step 2, see prompt in Table 2). These statements were then uploaded into the Concept System® Global MAX™ software (Concept Systems, Inc., Ithaca, USA, Copyright 2004–2020; all rights reserved), which creates sorting cards and rating worksheets for each participant. Participants were asked to sort these into groups of cards that seemed related to each other (Step 3), and to record a name for each grouping (or pile). Once done sorting, participants completed the rating worksheet, where they rated the importance of each statement to their health (Step 3, see prompt and scale in Table 2), and also completed a background and feedback form.

**Figure 1:**
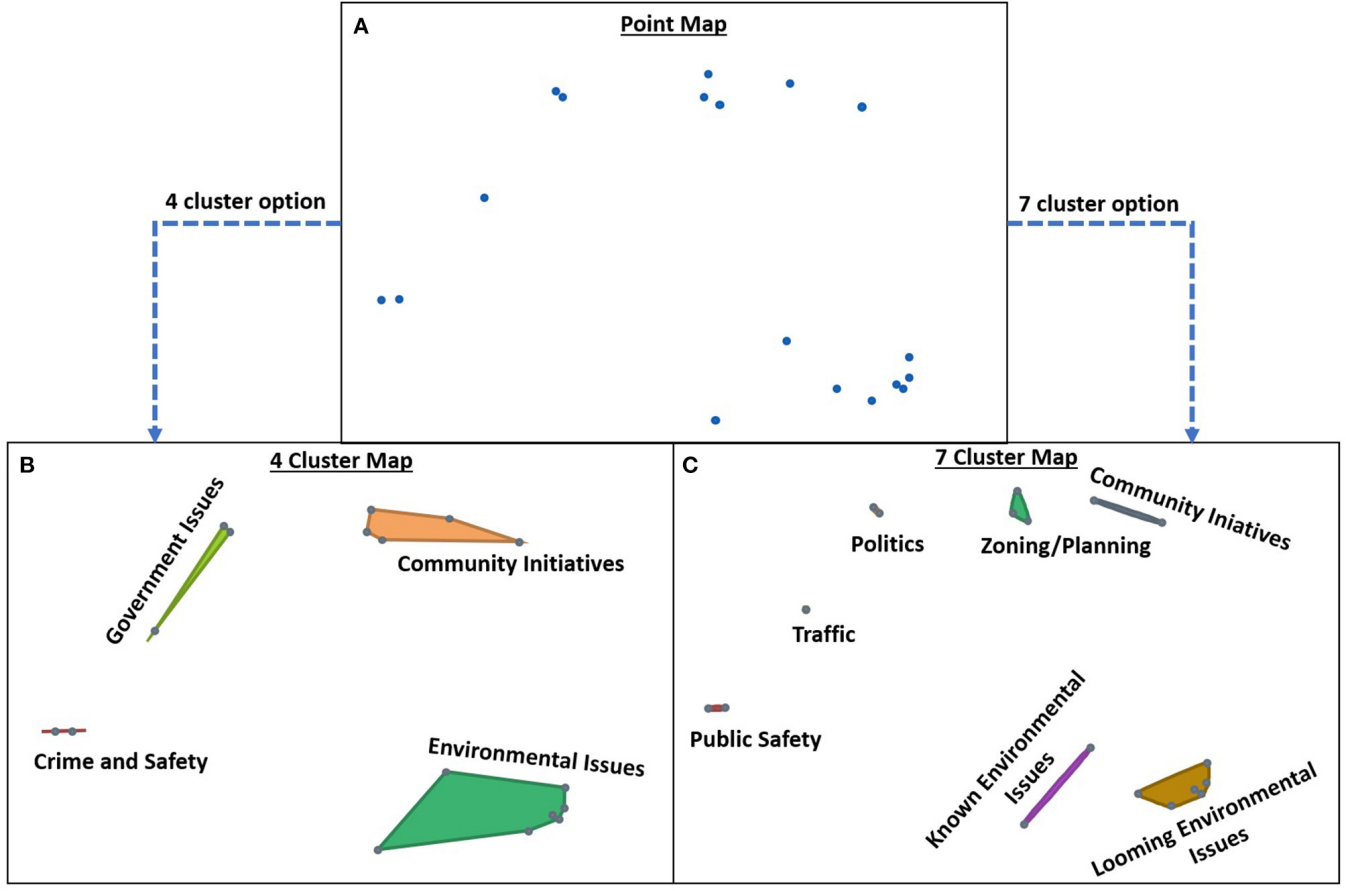
Point Map and Cluster Maps. The Point Map **(A)** represents each statement generated by participants, with statements that were frequently sorted together placed closer together on the map. Using hierarchical cluster analysis, the points are grouped into different arrangements of clusters and possible cluster labels. Two cluster arrangements are depicted here **(B,C)**, with different labels representing pile names provided by participants during the sorting phase. See Figure 2D for the cluster rating map, which depicts the average rating for each cluster in the final concept map chosen by Community D.

**Figure 2:**
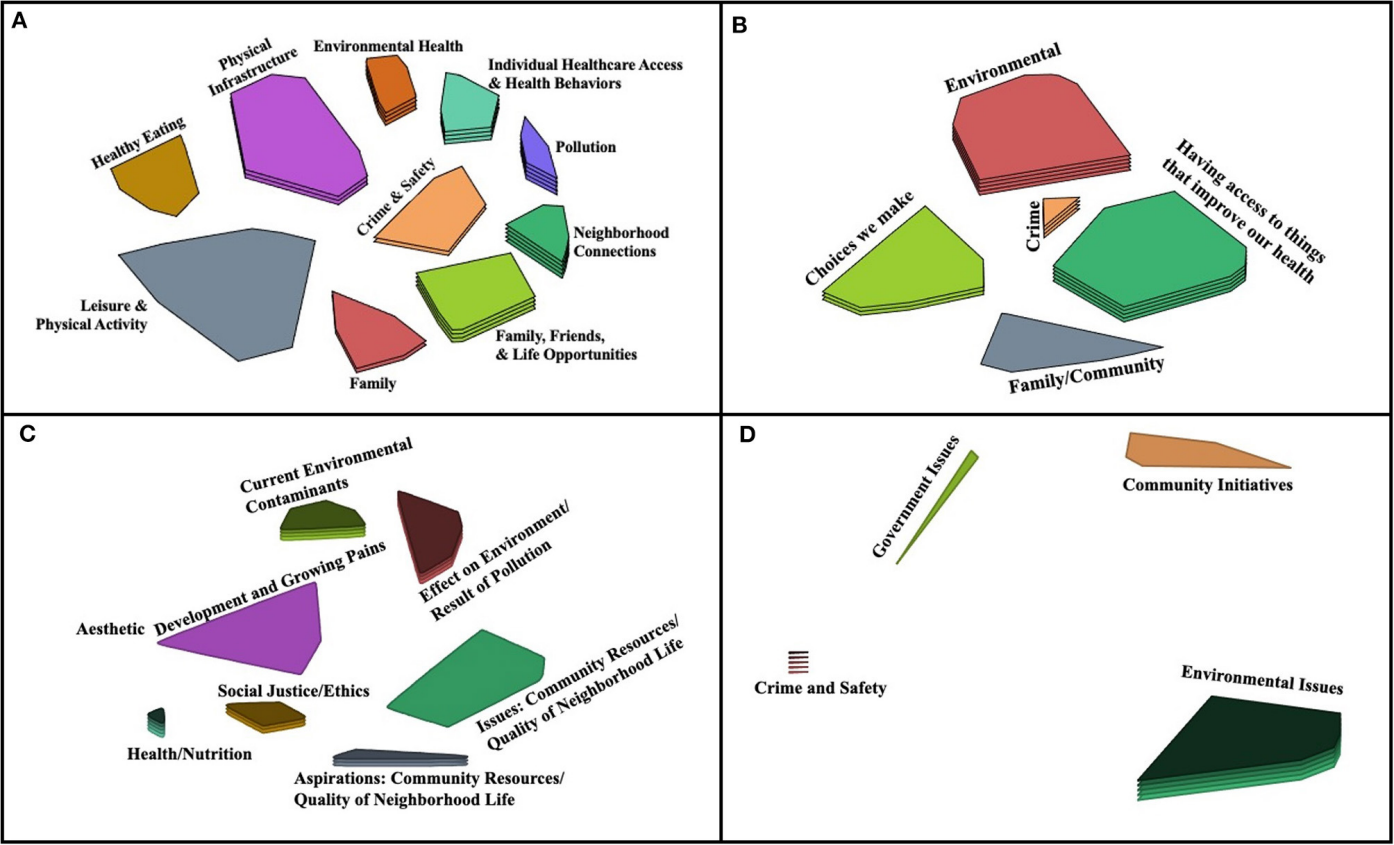
Final Concept Maps by Community. The cluster rating maps depict the final concept map chosen by each community (**A**: Community A; **B**: Community B; **C**: Community C; and **D**: Community D). Clusters in close proximity are more closely related than distant clusters. In **(C)**, the Aesthetic cluster represents a single statement that the community did not perceive as belonging with any of the other clusters and so chose to leave separate.

Between sessions, Emory staff entered participant sorting and rating data into the Concept Systems software, which created visual representations of the ideas generated by participants by placing each statement as a point on a two-dimensional map using non-metric multidimensional scaling (Step 4). Statements that were frequently sorted together were placed closer together on the point map, and the software grouped them into clusters using hierarchical cluster analysis, with each cluster representing a discrete theme that emerged from how participants sorted the brainstormed statements. The clusters that are closer to each other are more closely related conceptually, and vice versa (see Figure 1). The software also provided rating and bridging data for each statement and cluster (see Supplementary File A). Rating values illustrate the perceived importance of each issue or theme to the community's health and are represented visually by the cluster rating map (see Figure 2). Bridging values, which range from 0 to 1, represent the cohesiveness of the clusters and can be found in Supplementary File A. A cluster with a higher average bridging indicates that its statements “bridged” to other areas of the map and is therefore interrelated with other clusters.

The software produced multiple maps with different cluster arrangements and possible cluster labels using the pile names provided by participants during the sorting phase (Figure 1), and calculates a stress value for each map (**Table 4**). Stress values are a goodness of fit indicator that can be used to assess how well the map aligns with the participants' data, with lower values indicating better fit with the raw data. The average stress value for concept mapping projects is 0.28 (range 0.17–0.34, 95% CI [0.27, 0.29]) (14). Before the second session, staff met with the community liaison(s) and presented maps with different cluster arrangements to determine which map best represented the community's knowledge and experience, including the preferred labels for each cluster using the names provided by the participants. At the second workshop the community collectively chose the most appropriate label for each cluster and agreed on a final concept map to represent their community (Step 5). In session feedback, over 75% of participants felt that their final map accurately depicted their community's concerns very well and that the concept maps were very easy to understand. The final map was utilized (Step 6) to guide the community through the remaining components of the full program, which are described elsewhere (11). The results and discussion of this paper present further interpretation and utilization (Steps 5 and 6) of these Atlanta communities' concept maps, with the goal of exploring and understanding how communities perceive their exposome and using that definition to inform exposome science.

## Results

We completed concept mapping with a total of 118 community members in four communities. The participating community members were from four self-identified geographic communities located in distinct areas of metro-Atlanta and will be referred to as Community A, B, C, and D as specific neighborhoods have been redacted to protect confidentiality. Demographic information was collected from participants who completed concept mapping steps two and three at the first session (Table 3). We encouraged the same participants to attend both sessions, but as Table 3 demonstrates, this was not always possible.

**Table 3:** Demographics of roadshow workshop participants by community.

	**Community A**	**Community B**	**Community C**	**Community D**	**All participating communities**
Unit of Identity	Contiguous neighborhoods in Atlanta	Contiguous neighborhoods in small municipality	District within small municipality	Neighborhood in unincorporated county	
Total unique participants^a^	40	23	25	30	118
Attended Session 1 (Steps 2 & 3)	35	21	16	7	79
Participated in Step 3^b^	28	21	16	29	94
Attended Session 2 (Steps 5 & 6)	31	22	19	27	99
**Demographics**	*n* = 28 (%)	*n* = 21 (%)	*n* = 16 (%)	*n* = 29 (%)	94 (%)
**Sex**					
Female	20 (71.4)	14 (66.7)	7 (43.8)	19 (65.5)	60 (63.8)
Male	8 (28.6)	7 (33.3)	9 (56.3)	10 (34.5)	34 (36.2)
**Age**				*n* = 7^b^	*n* = 72^b^
18–34	7 (25.0)	5 (23.8)	7 (43.8)	0 (0)	19 (26.4)
35–49	2 (7.1)	4 (19.0)	1 (6.3)	0 (0)	7 (9.7)
50–64	10 (35.7)	6 (28.6)	0 (0)	2 (28.8)	18 (25.0)
65+	9 (32.1)	4 (19)	7 (43.8)	5 (71.4)	25 (34.7)
Unknown	0 (0)	2 (9.5)	0 (0)	22^b^	2 (2.1)
**Race**				*n* = 7^b^	*n* = 72^b^
Black	25 (89.3)	14 (66.7)	13 (81.3)	7 (100)	59 (81.9)
White	1 (3.6)	6 (28.6)	2 (12.5)	0 (0)	9 (12.5)
Other	2 (7.1)	1 (4.8)	0 (0)	0 (0)	3 (4.2)
Unknown	0 (0)	0 (0)	1 (6.3)	22^b^	1 (1.4)
**Education**					
High school graduate or GED	10 (35.7)	2 (9.5)	1 (6.3)	0 (0)	13 (13.8)
Some college/Trade school/associates degree	9 (32.1)	7 (33.3)	6 (37.5)	10 (34.5)	32 (34.0)
College graduate	7 (25.0)	5 (23.8)	5 (31.3)	8 (27.6)	25 (26.6)
Post-graduate degree	1 (3.6)	7 (33.3)	4 (25.0)	11 (37.9)	23 (24.5)
Unknown	1 (3.6)	0 (0)	0 (0)	0 (0)	1 (1.1)
**Income**					
<$10,000	5 (17.9)	1 (4.8)	4 (25.0)	0 (0)	10 (10.6)
$10–25,000	12 (42.9)	3 (14.3)	1 (6.3)	1 (3.4)	17 (18.1)
$25–50,000	5 (17.9)	1 (4.8)	1 (6.3)	1 (3.4)	8 (8.5)
$50–75,000	0 (0)	7 (33.3)	6 (37.5)	5 (17.2)	18 (19.2)
$75,000 or more	1 (3.6)	7 (33.3)	4 (25.0)	19 (65.5)	31 (33.0)
Unknown	5 (17.9)	2 (9.5)	0 (0)	3 (10.3)	10 (10.6)

a *We limited the number of attendees after encountering challenges conducting concept mapping with too many people in Community A*.

b *Due to low turnout at Session 1, Community D invited additional residents to participate in Sorting and Rating online before Session 2. Software limitations prevented us from collecting all demographic data from online participants, represented as “unknown.” These responses are not included in the percent totals*.

Participants in the four communities differed on various characteristics, but the majority were African American women over the age of 50. Attendees were mostly representative of their relevant census tracts, though more older and female residents participated as compared to their relative census tracts, and in all but one community household income was higher than the tract's median (15). This remains true when comparing to the larger Atlanta-metro area, in which the proportion of older females that participated in the Workshops was greater than that in the Atlanta region (15). Notably, while largely reflective of their respective communities, many more of our participants were Black than in the greater Atlanta-metro area (82 and 39%, respectively), and also more highly educated (15). Roadshow participants had both higher and lower household incomes, with more participants reporting incomes below $10,000 (Communities A and C) and above $75,000 (Community D) than the region as a whole (15).

### The Concept Maps

The final concept maps, or “cluster rating maps” selected by each of the four communities are shown in Figure 2, with the number of statements and clusters that comprise each map detailed in Table 4. Clusters are comprised of statements that were frequently sorted together, with cluster labels chosen by the community. Clusters that are closer to each other are more closely related, conceptually, and vice versa. There is more space between the clusters in Community D's map than the other communities, which could be due to the small number of participants and statements generated by this community during Session One. On each map, the number of layers of a cluster indicate the average cluster rating, with more layers indicating a higher rating (Figure 2). Higher rated clusters contain statements perceived as most important to the community's health, with the highest rated cluster of each community identified in Table 4. Average cluster bridging values for all communities ranged from 0.10 to 0.98, indicating that some clusters were highly cohesive, while others were highly interrelated with the other clusters (Table 4). The final stress value of the maps ranged from 0.1552 to 0.3420, with an average of 0.2494 (Table 4), which falls within the average range for concept mapping projects (mean 0.28, range 0.17–0.34, 95% CI [0.27, 0.29]) and indicates goodness of fit (14). Community D's final map had the lowest stress values, indicating a good fit while Community A's was higher than average indicating lower goodness of fit.

**Table 4:** Final concept map descriptors.

	**Community A**	**Community B**	**Community C**	**Community D**
# of statements	96	91	43	18
# of clusters	10	5	7	4
Cluster rating range	4.18–4.69	3.79–4.13	6.74–8.84	8.02–9.03
Highest rated cluster	Pollution	Environmental	Health/Nutrition	Public Safety
Bridging range	0.12–0.75	0.22–0.61	0.10–0.83	0.29–0.98
Stress value	0.3420	0.2620	0.2385	0.1552

### Identifying the Community Definition of the Exposome: Interpretation and Utilization

While each of the four communities differed in the number of clusters identified, ranging from four to 10 clusters, across all the communities we were able to identify five major themes: conventional environmental issues, the built environment, social relationships, crime and safety, and individual health and health behaviors. Table 5 presents each major theme and the individual community clusters of which it is comprised. Out of the 27 clusters identified across all four communities, 24 fit into one of the five major themes. The remaining three clusters reflected ideas that did not fit into one of the five major themes, yet the nature of the concept mapping approach indicates that when ideas form a distinct cluster, they are important by their very nature, and so they are included as an “Other General External” theme. Examples of the statements that comprise each theme can be found in Table 5. The full list of statements that form each cluster are available in Supplementary File A. In the Discussion, we further interpret and explore these themes within the context of existing exposome and public health research and discuss their implications for exposome research.

**Table 5:** Concept mapping clusters by community and theme.

	**Cluster names by community** ^a^			
**Major theme** ***(Example statements)***	**Community A**	**Community B**	**Community C**	**Community D**
**Conventional environmental issues** *(“chemicals and fumes in the home,” “release of toxins in the air,” “safe drinking water,” “pollution”)*	▪ Environmental Health and Related Illness ▪ Pollution	▪ Environmental	▪ Current Environmental Contaminants ▪ Effect on Environment/ Result of Pollution	▪ Environmental Issues
**Built environment** *(“access to quality health resources and services,” “traffic,” “accessing fresh fruits and vegetables,” “vacant/abandoned properties,” “lack of park space”)*	▪ Physical Infrastructure	▪ Having Access to Things that Improve our Health	▪ Development and Growing Pains ▪ Aesthetic ▪ Issues & Aspirations: Community Resources/ Quality of Neighborhood Life^b^	▪ Community Initiatives
**Social relationships** *(“youth involvement in community,” “family violence,” “being a caretaker for a family member,” “having a good parent as a child,” “community volunteering opportunities”)*	▪ Family ▪ Family, Friends, and Life Opportunities ▪ Neighborhood Connections	▪ Family/ community	▪ Issues & Aspirations: Community Resources/ Quality of Neighborhood Life^b^	
**Crime & safety** *(“gun violence,” “crime,” “security/safety,” “bullying,” “drugs in the community”)*	▪ Crime & Safety	▪ Crime	▪ Issues: Community Resources/ Quality of Neighborhood Life	▪ Crime & Safety
**Individual health & health behaviors** *(“asthma,” “walking,” “the food I eat,” “health literacy”)*	▪ Healthy Eating ▪ Leisure and Physical Activity ▪ Individual healthcare access and health behaviors	▪ Choices we make	▪ Health/Nutrition	
**Other general external exposures**	▪ Family, Friends, and Life Opportunities Example statements: “Education,” “Income”		▪ Social Justice/Ethics Example statements: “Flaws, practices of a racist society,” “Poverty”	▪ Government Issues Example statements: “Lack of responsiveness from local government,” “Lack of transparency”

a *Clusters that contain statements spanning more than one topic are repeated*.

b *Represents two clusters: “Issues: Community Resources/Quality of Neighborhood Life” and “Aspirations: Community Resources/Quality of Neighborhood Life”*.

## Discussion

We used concept mapping during the Exposome Roadshow to understand how communities define their exposome and to support communities in addressing their environmental health concerns. We explored the community-identified issues to learn how their perspective and lived experience can inform exposome science. We identified five major themes across the four communities (Table 5), and what follows is our exploration of each theme within the context of current public health and exposome science literature. We conclude with recommendations to enhance exposome science by including the community perspective, which we believe will lead to greater public health impact.

### Conventional Environmental Concerns

All four communities identified at least one cluster that was related to conventional environmental concerns (see Table 5). These environmental concerns were rated as extremely important by all four communities, as reflected by either the highest or second highest rating in each community (Table 4 and Figure 2A “Pollution,” **B** “Environmental,” **C** “Effect on Environment/Result of Pollution,” and **D** “Environmental Issues”). This community-identified theme is well-represented in the academic definition of the exposome as conventional environmental concerns form the basis for exposome definitions and exposome research. For example, exposome definitions refer to “toxicants in the general environment” (1), “exposures from the environment” (4), and “chemical pollutants” (16). Furthermore, exposome tools and methodologies are typically based on traditional exposure science (1, 4). Newer approaches to exposome science such as the public health exposome (6), the socio-exposome (7), and the urban exposome (8) also build upon the foundation of traditional exogenous environmental exposures and exposure science, while adding new layers and methods from the social sciences.

### Built Environment

All four communities identified at least one cluster related to the built environment (see Table 5), with one community rating it as the second most important to their health (see Figure 2B “Having access to things that improve our health”). These clusters included a wide range of statements (Table 5), which reflect a variety of built environment factors that are also supported by the literature (17–24). Given the built environment's impact on health, supported by both the community's lived experience and the scientific literature, exposome science needs to incorporate the built environment in order to accurately characterize the exposome. Traditionally, few exposome researchers have specifically included the built environment in their definitions of the external exposome, although components of the built environment have been referenced: the urban-rural environment (3), population density and sanitation (25), and location and green space (26). More recent frameworks such as the socio-exposome and the urban exposome (7, 8) explicitly reference the built environment.

When operationalizing the exposome concept, built environment factors are sometimes measured, often forming the source of conventional environmental exposures, such as roads and zoning that lead to traffic, industrial, or solid waste pollution (27), and urban settings or older homes that contain heavy metals (28). Data science tools used in exposome science include geographic information systems (GIS) that can be used to map various features of the built environment, such as location (city center, suburban, industrial, and rural) (29), access to affordable food (30), building density, public transportation, and greenspace (8). These features can then be combined with traditional exposure measurements to characterize the exposome.

### Social Relationships

Three of the four communities identified a cluster that was related to social relationships (see Table 5). Community-generated statements in these clusters focus on both community connectedness as well as interpersonal relationships while also considering some of the responsibilities or impacts of these relationships (Table 5). In Community A, community connectedness was the second most important issue affecting their health (see Figure 2A “Neighborhood Connections”). Aligned with this community definition of the exposome, there is ample evidence that interpersonal relationships have a cumulative impact on health across the lifetime (31). In fact, social support, social networks, and social capital have long been factors considered in the fields of social psychology and public health (32–34).

Some exposome researchers have included aspects of this community-identified theme in their definition and operationalization of the exposome. Wild (3) includes social influences and social capital as further external exposures in his revised definition of the exposome, while Rappaport (35) included psychosocial stress. Miller and Jones (4) also specifically reference how interactions with family, community, and society affect one's behavior in their definition of the exposome, and the public health exposome considers social support as a protective factor (6). In the HELIX study, these factors were operationalized by measuring social capital of the family and stress of the mother (36).

### Crime and Safety

All four communities had a cluster related to crime and safety (Table 5). In two communities (A and B), the crime cluster is located in the middle of the concept map (Figures 2A,B), and in communities B, C, and D it had a relatively high bridging score, demonstrating the interconnectedness of how crime and safety are perceived alongside other community exposures (Supplementary File A). “Public Safety” was rated as the most important issue affecting the health of Community D (Table 4). Furthermore, being identified by community members as its own separate category indicates its perceived importance as an environmental factor among residents of these metro-Atlanta communities.

Other community-engaged projects seeking to identify residents' environmental health concerns have similarly found that community members see crime and safety as one of the biggest environmental risks in their communities (37, 38). Crime is often considered in research around the impact of the built environment and structural and social determinants on health (39–42). A systematic review and a national survey both found associations between community violence and a variety of physical and mental health outcomes among children and youth (43, 44), including one study that found that air pollution predicted an asthma diagnosis only in children exposed to community violence (45).

However, crime is rarely, if ever, specifically named in the field of exposome science and research. While it fits within the broad exposure concepts that are sometimes posited and assessed in exposome research, such as the urban-rural environment (3) or psychosocial stressors (35), these concepts are not operationalized to include crime or safety. Crime is identified in the public health exposome as an exposure within the “social environment” (6) and within the socio-exposome's community level (7). Yet, neither framework's emphasis is as strong as the importance identified by these communities.

### Individual Health and Behaviors

Three of the four communities included clusters that related to individual health and behaviors (see Table 5). Community-generated statements in these clusters included factors related to individual health, nutrition, and health behaviors (Table 5). “Health/Nutrition” was rated as the most important to Community C's health (Table 4), but these issues were prioritized less in Community A and B (Figure 2A “Healthy Eating,” “Leisure and Physical Activity” and **B** “Choices we make”). These personal health factors are more typical in definitions and operationalizations of the exposome concept, including Wild's original definition (1) which specifically names lifestyle factors as an environmental exposure. Much like this community-identified theme, diet is also specifically included in exposome definitions (2–4) and studies (29, 36).

### Other General External Exposures

Three of the participating communities each identified an additional cluster that represented other external factors that affect their health: “Family, Friends, and Life Opportunities,” “Social Justice/Ethics,” and “Government Issues” (see Table 5). While these clusters didn't appear in multiple communities to create an overarching theme, their identification as distinct clusters reflect their importance to the specific community. These factors provide examples of the complex and interconnected systems that make up the general external exposome (46) and the socio-political conditions found in the socio-exposome framework (7). Often referred to broadly as social factors or stressors, these conditions have been widely linked to health outcomes (47, 48). And, importantly, racism has been declared a public health crisis (49). However, these factors have only been included in some definitions and studies of the exposome. For example, the public health exposome (6) defines the social environment to include descriptors of social/economic conditions such as poverty, crime, racial segregation, and unemployment and the urban exposome (8) considers factors such as ethnicity, social class, and family income.

Huang et al. (50) reviewed the methods of 31 studies on the cumulative effects of social stressors and environmental chemicals and found that studies operationalized social stressors by measuring factors such as socioeconomic status (SES), race, and education, as well as some of the community-identified themes described here, including crime and neighborhood features. Senier et al. (7) propose levels of measurement and data sources to operationalize the socio-exposome beyond these social environment factors, adding the political environment (e.g., civic participation) and government policies (e.g., history of discrimination and segregation). Furthermore, Zota and VanNoy (51) explicitly call upon environmental health scientists to consider the intersection of racism with other systems of oppression (e.g., sexism) and offer data analytic strategies for advancing intersectionality in exposome research. Outside of exposome science, social epidemiologists and economists offer methods to measure social inequities on multiple levels, including, for example, discrimination (52), racial/ethnic segregation and racialized economic segregation (53), and SES (54–56).

### How Roadshow Communities' Exposome Definition Informs Exposome Science and Action

The Roadshow communities identified a range of determinants of health that are broadly supported by the literature, some of which are captured by traditional definitions of the exposome. The Roadshow created an opportunity to further extend the definition to include the built environment, social relationships, and particularly crime and safety that are less often included or measured in traditional exposome science. This emphasis may reflect the fact that the Roadshow participants were predominantly older Black/African-American women and two of the communities had disproportionately low incomes (Table 3). Given that the Black population continues to experience striking health disparities that are unexplained by socioeconomic position (57, 58), the factors identified in their exposomes and the priority given to these factors likely reflect the shared determinants of health experienced by communities that face systemic injustices. For example, they prioritized environmental exposures and pollutants, reflecting the environmental injustices experienced by the participating communities. They also identified the importance of social capital and community support structures, which play an important role in African-American and low-income communities, particularly among women (59) and older adults (60), providing some protection from crime and other social stressors such as racism (61, 62). The emphasis on crime across communities may also reflect the urban location of the Roadshow communities. The intersection of race, gender, income, and place experienced by these communities underscores the importance of incorporating their perspectives into exposome science.

While some commentaries and discussions about the exposome have grown to acknowledge many of the factors that the Roadshow communities experience, the methods used to measure these factors rarely capture the specific exposures detailed in the communities' concept maps. Although biomarkers of stress and discrimination, including epigenetic changes, have been documented and are available for incorporation into exposome science (63–65), the operationalization of these factors and the methods to study their cumulative impact have not been routinely incorporated into exposome science. Relevant to Rappaport's (2) critique of traditional environmental and epidemiological methods that fragment environmental risks, these communities experience multiple types of environmental exposures collectively, and we need to study them as such. Specifically, factors related to the built environment, social relationships, crime and safety, and social factors such as poverty, racism, and political structures need to be included in studies of the exposome to adequately capture the cumulative environmental risks individuals and communities experience and prioritize. This inclusion is necessary if exposome science is to be translated into actions that reduce environmental injustices (7). This community definition helps illuminate the “unknown” risk factors that contribute to health outcomes (66) by highlighting the factors that interact with traditional environmental exposures to impact health. In other words, returning to Wild's expanded definition of the exposome (2), the community has identified factors in both the specific and general external environments, highlighting that characterizations of the exposome and the associated causal pathways need to expand beyond the endogenous metabolome. Therefore, we recommend the incorporation of this community definition into exposome science, while also emphasizing that communities may experience aspects of the exposome differently. For example, in another community, the built environment may have a positive impact on the community's exposome while crime may have a low impact. By incorporating these community-identified themes, the exposome definition works across communities with the flexibility to increase the benefits of exposome science in specific communities. Ultimately, to truly capture this wide variety of exposures, transdisciplinary research will be required (27).

One such discipline is community-engaged research. As recommended by others (6, 7), community-engaged research approaches can improve exposome science and increase its benefits. Working together to learn a community's lived experience is essential to accurately capture and measure their exposome, document inequities, and identify how to translate the findings into action (7). In the current approach, concept mapping provided one possible avenue to capture the specific factors experienced by these communities and their perceived importance, while their placement on the maps elucidates the interconnectedness of factors. The priorities that a specific community places on the components of the exposome can identify the scientific questions and methodology. As similarly noted by Zota and VanNoy (51), these lived experiences and their intersections are not captured by the majority of current approaches to exposome science, which do not capture concepts related to disproportionate exposures traditionally experienced by communities at the margin (specifically Black, Indigenous, and people of color and lower income). This limitation continues to perpetuate a science that lacks inclusivity and is void of true lived experiences. In practice, community-engaged research fundamentally embraces diversity of thought, creates opportunity for shared leadership and uplifts community understanding and expertise (67, 68). Using this perspective decentralizes science construction from traditional colonial perspectives to one that includes the knowledge of communities exposed to the multiplicities of environmental harms (69, 70). The community definition of the exposome that emerged from these four Atlanta-based communities reflects the need for the approach recommended as part of the socio-exposome framework, specifically the need to include and measure place-specific exposures, especially when hazards are concentrated in particular communities, and to partner with affected communities throughout the research process and its translation into action and policy (7). This type of paradigm shift allows for the creation of a more inclusive, comprehensive, and practical understanding of the exposome and its translation to tangible real-world benefits.

### Limitations

The Exposome Roadshow and concept mapping allowed us to successfully define four distinct community exposomes and identify major cross-cutting themes. However, this process is not without its limitations. The Roadshow approach itself presented biases that may have influenced results. First, the exposome concept originated in the environmental health sciences, and HERCULES is funded as an NIEHS environmental health core research center. Communities are made aware of the environmental health background of the Center when applying to participate in the Roadshow, which could explain why each community included clusters that were related to conventional environmental concerns and why these clusters were, on average, rated as extremely important by community members. We tried to address this bias in the exposome analogy presentation in Session One, which encouraged participants to take a holistic approach to the exposome and resulted in the inclusion of many factors beyond conventional environmental concerns. Notably, the staff conducting the Roadshow were White, and Emory is a predominantly White institution, contrasting with the predominantly Black participants and communities. This racial difference may have suppressed some issues, including racism and social justice. We foresaw this potential bias and so asked all participants to brainstorm, sort, and rate individually and anonymously. Our non-white SAB members also foresaw this issue, and several helped facilitate the Roadshow sessions. Yet, issues of race and social justice only emerged as a distinct cluster in one community. However, aligned with the qualitative tenets of concept mapping, its singular emergence is still worth noting and has been included in our community definition of the exposome. Lastly, the Roadshow participants generally represented their census tracts and communities, but the demographics skewed slightly toward older female participants who may have different perspectives than both younger residents and male residents. The liaisons also attempted to recruit other community stakeholders, such as business and non-profit leaders who would have valuable knowledge to contribute but were largely unsuccessful. In future workshops, we will expand our efforts to include more representative stakeholders. This community definition of the exposome represents the experience of four distinct community groups in one Southeast US city, and may not be generalizable to other communities. However, the insights gained through the Roadshow can provide academic scientists with a breadth of factors that should be considered when conducting exposome science. Further this approach is shared as one community-engagement model/method that can be useful for obtaining a more comprehensive understanding of a community-specific exposome.

The concept mapping approach also had its limitations, especially given the iterative nature of our process. For example, after conducting the workshops with Communities A and B we changed the brainstorming question in order to focus the statements more on the communities' health and set the community up to take action on their concerns (Table 2). Additionally, after receiving almost 150 statements from 35 people during Community A's workshop (of which they chose to include 96 in their final map) we limited the number of statements per participant to five instead of seven and the number of participants from 35 to 25. This high number of statements and participants is above average for concept mapping projects (14) and could explain the high stress level of Community A's concept map. Yet, the average stress level of the four concept maps is similar to other studies (14). Furthermore, we did not use the stress value to select the final maps. Instead, the community liaisons chose the cluster arrangements and then the community agreed as to which map best represented them and reflected their exposome. Lastly, our concept mapping approach asked participants to disentangle and sort factors that are inherently interrelated, such as social factors, crime, and the built environment. However, we do not lose these nuances as the maps still depict the relationships and interconnectedness between clusters and statements in the positioning of clusters on the maps and through the bridging scores. In all, concept mapping stays true to the complexities of the lived experience and the exposome while also disentangling some of those complexities to facilitate focused action and bring distinct scientific disciplines together.

## Conclusions

The exposome is a complex concept that many scholars have worked for years to elucidate. As such, its definition and operationalization continue to evolve. We sought to contribute to that evolution, filling an important gap in existing exposome approaches that lack a community perspective. Since the inception of the HERCULES Exposome Research Center, our SAB and community partners have told us that the concept behind exposome science reflects their lived experience, which is a complex interplay of multiple exposures across the lifetime. When asked about the exposome concept in the Roadshow evaluation, one participant stated, “it is spot on.” Yet, exposome science has yet to truly capture this complexity.

Our approach highlights the value of the lived experience and, importantly, provides a pathway for community knowledge into exposome science, which has largely excluded these perspectives. The community's exposome presented here provides a framework that academic scientists can use to incorporate different scientific disciplines and approaches into their study of the exposome. Incorporating the community's definition (including its priorities) into exposome science is essential to reach the full potential of the exposome concept. Partnering with social scientists and environmental justice communities in exposome science will increase the likelihood that the disproportionate exposures experienced by these communities will be measured, leading to improved scientific outcomes and tangible public health impact.

## Data Availability Statement

The original contributions presented in the study are included in the article/Supplementary Material, further inquiries can be directed to the corresponding author/s.

## Author Contributions

DW, EL-S, LY, MK, MH, MP, KB, and YN contributed to conception and design of the program. EL-S and MP implemented the program and performed analysis. EL-S, MP, and MST wrote the first draft of the manuscript. All authors contributed to manuscript revision, read, and approved the submitted version.

## Funding

The HERCULES Exposome Research Center is funded by the National Institutes for Environmental Health (P30 ES019776). The Rollins Endowment at the Emory University Rollins School of Public Health open access publication fees.

## Conflict of Interest

The authors declare that the research was conducted in the absence of any commercial or financial relationships that could be construed as a potential conflict of interest.

## Publisher's Note

All claims expressed in this article are solely those of the authors and do not necessarily represent those of their affiliated organizations, or those of the publisher, the editors and the reviewers. Any product that may be evaluated in this article, or claim that may be made by its manufacturer, is not guaranteed or endorsed by the publisher.

## References

[1] Wild CP. Complementing the genome with an “exposome”: the outstanding challenge of environmental exposure measurement in molecular epidemiology. Cancer Epidemiol Biomark Prevent. (2005) 14:1847–50. 10.1158/1055-9965.EPI-05-0456 16103423

[2] Rappaport SM Smith MT. Epidemiology. Environment and disease risks. Science. (2010) 330:460–1. 10.1126/science.1192603 20966241 PMC4841276

[3] Wild CP. The exposome: from concept to utility. Int J Epidemiol. (2012) 41:24–32. 10.1093/ije/dyr236 22296988

[4] Miller GW Jones DP. The nature of nurture: refining the definition of the exposome. Toxicol Sci. (2014) 137:1–2. 10.1093/toxsci/kft251 24213143 PMC3871934

[5] Viet SM Falman JC Merrill LS Faustman EM Savitz DA Mervish N. Human Health Exposure Analysis Resource (HHEAR): A model for incorporating the exposome into health studies. Int J Hyg Environ Health. (2021) 235:113768. 10.1016/j.ijheh.2021.113768 34034040 PMC8205973

[6] Juarez PD Matthews-Juarez P Hood DB Im W Levine RS Kilbourne BJ. The public health exposome: a population-based, exposure science approach to health disparities research. Int J Environ Res Public Health. (2014) 11:12866–95. 10.3390/ijerph111212866 25514145 PMC4276651

[7] Senier L Brown P Shostak S Hanna B. The socio-exposome: advancing exposure science and environmental justice in a postgenomic era. Environ Sociol. (2017) 3:107–21. 10.1080/23251042.2016.1220848 28944245 PMC5604315

[8] Robinson O Tamayo I Castro Md Valentin A Giorgis-Allemand L Krog NH. The urban exposome during pregnancy and its socioeconomic determinants. Environ Health Perspect. (2018) 126:077005. 10.1289/EHP2862 30024382 PMC6108870

[9] Wiggins N. Critical pedagogy and popular education: towards a unity of theory and practice. Stud Educ Adults. (2011) 43:34–49. 10.1080/02660830.2011.11661602

[10] Wiggins N. Popular education for health promotion and community empowerment: a review of the literature. Health Promotion Int. (2012) 27 3:356–71. 10.1093/heapro/dar046 21835843

[11] Lebow-Skelley E Young L Noibi Y Blaginin K Hooker M Williamson D. Principles of Community Engagement 3rd ed. Washington DC: U.S. Department of Health and Human Services (In-Press).

[12] Trochim WM. An introduction to concept mapping for planning and evaluation. Evaluation Program Plan. (1989) 12:1–16. 10.1016/0149-7189(89)90016-5

[13] Atlanta Regional Commission (ARC). Atlanta Region. (2019). Available online at: https://atlantaregional.org/

[14] Rosas SR Kane M. Quality and rigor of the concept mapping methodology: a pooled study analysis. Eval Program Plann. (2012) 35:236–45. 10.1016/j.evalprogplan.2011.10.003 22221889

[15] U.S. Census Bureau. Selected Characteristics of the Total and Native Populations in the United States: 2012-2016.

[16] Turner MC Nieuwenhuijsen M Anderson K Balshaw D Cui Y Dunton G. Assessing the exposome with external measures: commentary on the state of the science and research recommendations. Ann Rev Public Health. (2017) 38:215–39. 10.1146/annurev-publhealth-082516-012802 28384083 PMC7161939

[17] Roux AVD Merkin SS Arnett D Chambless L Massing M Nieto FJ. Neighborhood of residence and incidence of coronary heart disease. New Engl J Med. (2001) 345:99–106. 10.1056/NEJM200107123450205 11450679

[18] Renalds A Smith TH Hale PJ. A systematic review of built environment and health. Fam Community Health. (2010) 33:68–78. 10.1097/FCH.0b013e3181c4e2e5 20010006

[19] Gelormino E Melis G Marietta C Costa G. From built environment to health inequalities: An explanatory framework based on evidence. Prevent Med Rep. (2015) 2:737–45. 10.1016/j.pmedr.2015.08.019 26844145 PMC4721462

[20] Gascon M Vrijheid M Nieuwenhuijsen MJ. The built environment and child health: an overview of current evidence. Curr Environ Health Rep. (2016) 3:250–7. 10.1007/s40572-016-0094-z 27220615

[21] Salgado M Madureira J Mendes AS Torres A Teixeira JP Oliveira MD. Environmental determinants of population health in urban settings. A systematic review. BMC Public Health. (2020) 20:853. 10.1186/s12889-020-08905-0 32493328 PMC7271472

[22] Cobb LK Appel LJ Franco M Jones-Smith JC Nur A Anderson CA. The relationship of the local food environment with obesity: A systematic review of methods, study quality, and results. Obesity. (2015) 23:1331–44. 10.1002/oby.21118 26096983 PMC4482774

[23] Diez Roux AV Mair C. Neighborhoods and health. Ann NY Acad Sci. (2010) 1186:125–45. 10.1111/j.1749-6632.2009.05333.x 20201871

[24] Moyer R MacDonald JM Ridgeway G Branas CC. Effect of remediating blighted vacant land on shootings: a citywide cluster randomized trial. Am J Public Health. (2019) 109:140–4. 10.2105/AJPH.2018.304752 30496003 PMC6301418

[25] Buck Louis GM Sundaram R. Exposome: time for transformative research. Stat Med. (2012) 31:2569–75. 10.1002/sim.5496 22969025 PMC3842164

[26] Nieuwenhuijsen MJ Donaire-Gonzalez D Foraster M Martinez D Cisneros A. Using personal sensors to assess the exposome and acute health effects. Int J Environ Res Public Health. (2014) 11:7805–19. 10.3390/ijerph110807805 25101766 PMC4143834

[27] Cannon CEB. Towards convergence: how to do transdisciplinary environmental health disparities research. Int J Environ Res Public Health. (2020) 17:2303. 10.3390/ijerph17072303 32235385 PMC7177595

[28] Filippelli G Adamic J Nichols D Shukle J Frix E. Mapping the urban lead exposome: a detailed analysis of soil metal concentrations at the household scale using citizen science. Int J Environ Res Public Health. (2018) 15:1531. 10.3390/ijerph15071531 30029546 PMC6069257

[29] Vineis P Chadeau-Hyam M Gmuender H Gulliver J Herceg Z Kleinjans J. The exposome in practice: Design of the EXPOsOMICS project. Int J Hygiene Environ Health. (2017) 220:142–51. 10.1016/j.ijheh.2016.08.001 27576363 PMC6192011

[30] Jiao Y Bower JK Im W Basta N Obrycki J Al-Hamdan MZ. Application of citizen science risk communication tools in a vulnerable urban community. Int J Environ Res Public Health. (2015) 13:ijerph13010011-ijerph. 10.3390/ijerph13010011 26703664 PMC4730402

[31] Umberson D Crosnoe R Reczek C. Social relationships and health behavior across the life course. Ann Rev Sociol. (2010) 36:139–57. 10.1146/annurev-soc-070308-120011 21921974 PMC3171805

[32] Andrews G Tennant C Hewson D Schonell M. The relation of social factors to physical and psychiatric illness. Am J Epidemiol. (1978) 108:27–35. 685973

[33] Sarason IG Levine HM Basham RB Sarason BR. Assessing social support: The social support questionnaire. J Personal Soc Psychol. (1983) 44:127–39. 10.1037/0022-3514.44.1.12722486356

[34] McLeroy KR Bibeau D Steckler A Glanz K. An ecological perspective on health promotion programs. Health Educ Q. (1988) 15:351–77. 10.1177/109019818801500401 3068205

[35] Rappaport SM. Biomarkers intersect with the exposome. Biomarkers. (2012) 17:483–9. 10.3109/1354750X.2012.691553 22672124 PMC4763608

[36] Maitre L de Bont J Casas M Robinson O Aasvang GM Agier L. Human Early Life Exposome (HELIX) study: a European population-based exposome cohort. BMJ Open. (2018) 8:e021311. 10.1136/bmjopen-2017-021311 30206078 PMC6144482

[37] White BM Hall ES Johnson C. Environmental health literacy in support of social action: an environmental justice perspective. J Environ Health. (2014) 77:24–9. Available online at: https://www.jstor.org/stable/26330074 25185324

[38] Cohen AK Lopez A Malloy N Morello-Frosch R. Surveying for environmental health justice: community organizing applications of community-based participatory research. Environ Justice. (2016) 9:129–36. 10.1089/env.2016.0008

[39] Crear-Perry J Correa-de-Araujo R Lewis Johnson T McLemore MR Neilson E Wallace M. Social and structural determinants of health inequities in maternal health. J Womens Health (Larchmt). (2021) 30:230–5. 10.1089/jwh.2020.8882 33181043 PMC8020519

[40] Lorenc T Clayton S Neary D Whitehead M Petticrew M Thomson H. Crime, fear of crime, environment, and mental health and wellbeing: mapping review of theories and causal pathways. Health Place. (2012) 18:757–65. 10.1016/j.healthplace.2012.04.001 22542441

[41] Saelens BE Handy SL. Built environment correlates of walking: a review. Med Sci Sports Exercise. (2008) 40:S550–66. 10.1249/MSS.0b013e31817c67a4 18562973 PMC2921187

[42] Sullivan K Thakur N. Structural and social determinants of health in asthma in developed economies: a scoping review of literature published between 2014 and 2019. Curr Allergy Asthma Rep. (2020) 20:5. 10.1007/s11882-020-0899-6 32030507 PMC7005090

[43] Jackson DB Posick C Vaughn MG. New evidence of the nexus between neighborhood violence, perceptions of danger, and child health. Health Affairs. (2019) 38:746–54. 10.1377/hlthaff.2018.05127 31059369

[44] Wright AW Austin MK Booth C Kliewer WL. Systematic review: exposure to community violence and physical health outcomes in youth. J Pediatric Psychol. (2017) 42:364−78. 10.1093/jpepsy/jsw088 27794530

[45] Clougherty JE Levy JI Kubzansky LD Ryan PB Suglia SF Canner MJ. Synergistic effects of traffic-related air pollution and exposure to violence on urban asthma etiology. Environ. Health Perspect. (2007) 115:1140–6. 10.1289/ehp.9863 17687439 PMC1940095

[46] Andrianou XD Makris KC. The framework of urban exposome: Application of the exposome concept in urban health studies. Sci Total Environ. (2018) 636:963–7. 10.1016/j.scitotenv.2018.04.329 29729514

[47] Marmot M. Inequalities in health. N Engl J Med. (2001) 345:134–6. 10.1056/NEJM200107123450210 11450663

[48] O'Neill MS Jerrett M Kawachi I Levy JI Cohen AJ Gouveia N. Health, wealth, and air pollution: advancing theory and methods. Environ Health Perspect. (2003) 111:1861–70. 10.1289/ehp.6334 14644658 PMC1241758

[49] American Public Heath Association (APHA). Racism is a Public Health Crisis. (2021). Available online at: https://www.apha.org/topics-and-issues/health-equity/racism-and-health/racism-declarations

[50] Huang H Wang A Morello-Frosch R Lam J Sirota M Padula A. Cumulative risk and impact modeling on environmental chemical and social stressors. Curr Environ Health Rep. (2018) 5:88–99. 10.1007/s40572-018-0180-5 29441463 PMC5876145

[51] Zota AR VanNoy BN. Integrating intersectionality into the exposome paradigm: a novel approach to racial inequities in uterine fibroids. Am J Public Health. (2021) 111:104–9. 10.2105/AJPH.2020.305979 33211578 PMC7750596

[52] Krieger N. Methods for the scientific study of discrimination and health: an ecosocial approach. Am J Public Health. (2012) 102:936–44. 10.2105/AJPH.2011.300544 22420803 PMC3484783

[53] Krieger N Kim R Feldman J Waterman PD. Using the Index of Concentration at the Extremes at multiple geographical levels to monitor health inequities in an era of growing spatial social polarization: Massachusetts, USA (2010–14). Int J Epidemiol. (2018) 47:788–819. 10.1093/ije/dyy004 29522187

[54] Oakes JM Rossi PH. The measurement of SES in health research: current practice and steps toward a new approach. Soc Sci Med. (2003) 56:769–84. 10.1016/S0277-9536(02)00073-4 12560010

[55] Harper S Lynch J Meersman SC Breen N Davis WW Reichman ME. An overview of methods for monitoring social disparities in cancer with an example using trends in lung cancer incidence by area-socioeconomic position and race-ethnicity, 1992–2004. Am J Epidemiol. (2008) 167:889–99. 10.1093/aje/kwn016 18344513 PMC2409988

[56] Breen N Scott S Percy-Laurry A Lewis D Glasgow R. Health disparities calculator: a methodologically rigorous tool for analyzing inequalities in population health. Am J Public Health. (2014) 104:1589–91. 10.2105/AJPH.2014.301982 25033114 PMC4151930

[57] Williams DR Lawrence JA Davis BA. Racism and health: evidence and needed research. Ann Rev Public Health. (2019) 40:105–25. 10.1146/annurev-publhealth-040218-043750 30601726 PMC6532402

[58] Bailey ZD Krieger N Agénor M Graves J Linos N Bassett MT. Structural racism and health inequities in the USA: evidence and interventions. Lancet. (2017) 389:1453–63. 10.1016/S0140-6736(17)30569-X 28402827

[59] Neergheen VL Topel M Van Dyke ME Sullivan S Pemu PE Gibbons GH. Neighborhood social cohesion is associated with lower levels of interleukin-6 in African American women. Brain Behav Immun. (2019) 76:28–36. 10.1016/j.bbi.2018.10.008 30686334 PMC6370481

[60] Henderson H Child S Moore S Moore JB Kaczynski AT. The influence of neighborhood aesthetics, safety, and social cohesion on perceived stress in disadvantaged communities. Am J Community Psychol. (2016) 58:80–8. 10.1002/ajcp.12081 27573035

[61] Collins CR Neal ZP Neal JW. Transforming social cohesion into informal social control: Deconstructing collective efficacy and the moderating role of neighborhood racial homogeneity. J Urban Affairs. (2017) 39:307–22. 10.1080/07352166.2016.1245079

[62] Hailu EM Lewis TT Needham BL Lin J Seeman TE Mujahid MS. Longitudinal associations between discrimination, neighborhood social cohesion, and telomere length: the multi-ethnic study of atherosclerosis. J Gerontol Series A. (2021) 77:365–74. 10.1093/gerona/glab193 34282826 PMC8824602

[63] Ghezzi P. Environmental risk factors and their footprints *in vivo* – A proposal for the classification of oxidative stress biomarkers. Redox Biol. (2020) 34:101442. 10.1016/j.redox.2020.101442 32035921 PMC7327955

[64] Condon EM Holland ML Slade A Redeker NS Mayes LC Sadler LS. Associations between maternal experiences of discrimination and biomarkers of toxic stress in school-aged children. Maternal Child Health J. (2019) 23:1147–51. 10.1007/s10995-019-02779-4 31222595 PMC6660374

[65] Mulligan CJ. Systemic racism can get under our skin and into our genes. Am J Phys Anthropol. (2021) 175:399–405. 10.1002/ajpa.24290 33905118

[66] Rappaport SM Barupal DK Wishart D Vineis P Scalbert A. The blood exposome and its role in discovering causes of disease. Environ Health Perspect. (2014) 122:769–74. 10.1289/ehp.1308015 24659601 PMC4123034

[67] Rumal BB Coleman SE Roary Y. Engaging People with Lived Experience: Pre-Relationship to Relationship Building Assessment Tool and Resource Guide. Cambridge, MA. (2019).

[68] Coleman SE Byrd K Scaccia JP Stout S Schall M Callender S. Engaging Community Members with Lived Experience. Cambridge, MA (2017).

[69] Wallerstein N Duran B. Community-based participatory research contributions to intervention research: the intersection of science and practice to improve health equity. Am J Public Health. (2010) 100:S40–6. 10.2105/AJPH.2009.184036 20147663 PMC2837458

[70] Minkler M Garcia AP Rubin V Wallerstein N. Community-Based Participatory Research: A Strategy for Building Healthy Communities and Promoting Health through Policy Change. University of California, Berkely School of Public Health (2012).

