# Supplementary material for: Defining the Exposome Using Popular Education and Concept Mapping With Communities in Atlanta, Georgia
Source: Front Public Health. 2022 Apr 12;10:842539. doi: 10.3389/fpubh.2022.842539 (PMC9039048; doi:10.3389/fpubh.2022.842539)
Supplement: Supplementary file 1 [file Table_1.DOCX]

| **Community A: Cluster and Statement List** |  |  |
| --- | --- | --- |
| **Cluster Names and Statements** | **Average Cluster Rating (1-5)** | **Cluster Bridging Value** |
| **Pollution** | 4.69 | 0.27 |
| Air we breathe |  |  |
| Toxic chemicals in water |  |  |
| Chemicals and fumes in the home |  |  |
| Lack of environmental attention |  |  |
| **Neighborhood Connections** | 4.63 | 0.28 |
| Being a part of a group |  |  |
| Life choices |  |  |
| Neighborhood vacancies – no one living in homes with children |  |  |
| Safety |  |  |
| Youth involvement in community |  |  |
| Community |  |  |
| Crime |  |  |
| Neighborhood social connections |  |  |
| Housing |  |  |
| Community environment |  |  |
| Death of loved ones |  |  |
| **Environmental Health and Related Illness** | 4.53 | 0.17 |
| Respiratory problems |  |  |
| Air pollution |  |  |
| Hay fever |  |  |
| Poor water quality |  |  |
| Atmosphere: plays a huge part in overall physical, emotional, and social wellbeing |  |  |
| Asbestos and lead paint in homes |  |  |
| Rodents |  |  |
| **Family, Friends and Life Opportunities** | 4.53 | 0.19 |
| Children |  |  |
| Relationships with people |  |  |
| Friends |  |  |
| Responsibilities |  |  |
| Income |  |  |
| Education |  |  |
| Equality |  |  |
| Life growing up in a good community |  |  |
| Writing |  |  |
| Teachers |  |  |
| Career |  |  |
| Peace and positivity |  |  |
| Paying bills and needs of children |  |  |
| Growing up with love |  |  |
| Attending meetings in the community to keep informed |  |  |
| School community |  |  |
| Church |  |  |
| Parents feed us |  |  |
| Strong extended family connections |  |  |
| **Health Care, Access and Health Behaviors** | 4.52 | 0.12 |
| Airborne diseases |  |  |
| Asthma |  |  |
| Lupus disease |  |  |
| Heart disease |  |  |
| Access to medicine/doctors |  |  |
| Not drinking water |  |  |
| High blood pressure |  |  |
| Smoking cigarettes |  |  |
| Alcoholism |  |  |
| Personal fear about sickness and disease |  |  |
| Dental care/habits |  |  |
| Health insurance |  |  |
| **Physical Infrastructure** | 4.44 | 0.47 |
| Accessing fresh fruits and vegetables |  |  |
| Vacant/abandoned properties |  |  |
| Living in a wasteful community |  |  |
| Keeping dust down in the home |  |  |
| Secondhand smoke |  |  |
| Poor infrastructure |  |  |
| Clean water |  |  |
| Quality of building construction |  |  |
| **Crime and Safety** | 4.33 | 0.17 |
| Gun violence |  |  |
| Bullying |  |  |
| Drugs in the community |  |  |
| Gangs kids focused on safety and school |  |  |
| Involvement in positive activities and people |  |  |
| Thinking less stressful |  |  |
| Drugs in family |  |  |
| **Family** | 4.33 | 0.27 |
| Grandparents |  |  |
| Ill and elderly family members |  |  |
| Parents |  |  |
| Living with grandparents |  |  |
| Family violence |  |  |
| Military families |  |  |
| Extended support system |  |  |
| Absent parents |  |  |
| Stability |  |  |
| Having children at an early age |  |  |
| **Leisure and Physical Activity** | 4.19 | 0.61 |
| Walking |  |  |
| Outdoor activities |  |  |
| Traveling |  |  |
| Recess |  |  |
| Recreation |  |  |
| Vacation |  |  |
| No infrastructure for good education |  |  |
| Lifestyles |  |  |
| Park |  |  |
| Sports |  |  |
| Different organizations |  |  |
| **Healthy Eating** | 4.18 | 0.75 |
| Organic food |  |  |
| Eating healthy as a young adult |  |  |
| Fatty foods |  |  |
| Food deserts |  |  |
| Bad eating habits |  |  |
| Cooking methods |  |  |
| Poor development |  |  |
| Dental care/habits |  |  |
| Health insurance |  |  |
|  | | |

| **Community B: Cluster and Statement List** |  |  |
| --- | --- | --- |
| **Cluster Names and Statements** | **Average Cluster Rating**  **(1-5)** | **Cluster Bridging Value** |
| **Environmental** | 4.13 | 0.22 |
| Clean environment |  |  |
| Clean water |  |  |
| Water I drink |  |  |
| Release of toxins in the air |  |  |
| Harmful chemicals in environment |  |  |
| Clean air |  |  |
| Notification of water contamination |  |  |
| Access to clean, pesticide-free food |  |  |
| Water treatment |  |  |
| Contaminated ground water |  |  |
| Chemical smell |  |  |
| Soil pollution |  |  |
| Manufacturing pollution |  |  |
| Stagnant indoor air |  |  |
| Lung/breathing problems |  |  |
| Built environment |  |  |
| Occupational hazards/exposures |  |  |
| Lack of upkeep by property owners |  |  |
| Mold/mildew |  |  |
| Drinking water without fluoride |  |  |
| Smoking in public places |  |  |
| Indoor flooding |  |  |
| Not collecting rain water as a resource |  |  |
| **Having access to things that improve our health** | 4.10 | 0.41 |
| Access to quality health resources and services |  |  |
| The community I live in |  |  |
| Access to healthcare |  |  |
| Having adequate funds to support healthy lifestyle |  |  |
| Access to educational opportunities at all levels |  |  |
| Having or not having access to health insurance |  |  |
| Access to parks and playgrounds |  |  |
| Access to jobs |  |  |
| Access to mental health providers |  |  |
| Helping the community |  |  |
| Health awareness and knowledge |  |  |
| Getting the correct information to maintain a healthy lifestyle |  |  |
| Access to formal education |  |  |
| Livable centers (walkability, mobility, recreation, etc.) |  |  |
| Access to transportation |  |  |
| Access to community events |  |  |
| Access to informal education (e.g., workshops) |  |  |
| Lack of health education |  |  |
| PE classes in school |  |  |
| School programs that encourage group interaction as well as self-development |  |  |
| Healthy habits of people around me |  |  |
| Attending a good, safe school with great teachers and a lot of resources |  |  |
| Communication about opportunities to exercise |  |  |
| Church |  |  |
| Knowledge about how to exercise |  |  |
| Thinking classes |  |  |
| Yoga classes |  |  |
| **Crime** | 4.00 | 0.42 |
| High crime |  |  |
| Location |  |  |
| Gun violence |  |  |
| Poverty |  |  |
| **Choices we make** | 3.96 | 0.61 |
| The food I eat |  |  |
| Adequate sleep |  |  |
| Access to healthy, affordable fruits, vegetables, meat, grains, and fish |  |  |
| Healthy foods |  |  |
| Ability to spend time outside walking, working in yard, walking dogs |  |  |
| Eating a balanced meal 3 times a day |  |  |
| Willingness to care about health/take action |  |  |
| How to prepare/cook healthy food |  |  |
| Stress |  |  |
| Making healthier food choices |  |  |
| My job |  |  |
| Not having energy to do things |  |  |
| Blood pressure |  |  |
| Walking club |  |  |
| Lack of physical activities as an adult |  |  |
| Aggressive employers |  |  |
| Parents growing own food |  |  |
| **Family/Community** | 3.79 | 0.30 |
| Having a good parent as a child |  |  |
| A good circle of friends that was both supportive and critical |  |  |
| Having time |  |  |
| Positive self-esteem |  |  |
| Supportive family as an adult |  |  |
| Supportive home environment |  |  |
| Relationships with friends and loved ones |  |  |
| Social ties |  |  |
| Personality type |  |  |
| Financial status of family |  |  |
| Friendlier attitude |  |  |
| Volunteering |  |  |
| Sense of safety as a child |  |  |
| Marital status/having a partner |  |  |
| Being a caretaker for a family member |  |  |
| Playing with friends as a child |  |  |
| Having a child and being responsible for them |  |  |
| Church involvement in childhood |  |  |
| Divorce |  |  |
| Leaving family in early adulthood |  |  |

| **Community C: Cluster and Statement List** | | |
| --- | --- | --- |
| **Cluster Names and Statements** | **Average Cluster Rating**  **(1-10)** | **Cluster Bridging Value** |
| **Health/Nutrition** | 8.84 | 0.42 |
| Available (healthy) food options |  |  |
| Health literacy |  |  |
| Nutrition |  |  |
| **Effect on Environment/Result of Pollution** | 8.41 | 0.1 |
| Tainted water wells |  |  |
| Water pollution (to lakes) |  |  |
| Chemical attacks on the environment |  |  |
| Litter/pollution (trash and tires) |  |  |
| Pollution |  |  |
| Erosion |  |  |
| Unhealthy vegetation |  |  |
| Tree depletion |  |  |
| Wildlife displacement |  |  |
| Soil contamination |  |  |
| **Current Environmental Contaminants** | 8.01 | 0.25 |
| Warehouses (truck exhaust pollution) |  |  |
| Leaching and burning landfills |  |  |
| Storm water |  |  |
| Pesticide poisoning |  |  |
| Poor air quality |  |  |
| **Social Justice/Ethics** | 7.96 | 0.28 |
| Flaws, practices of racist society |  |  |
| Injustice leading to poor health outcomes |  |  |
| Distrust caused by marketing and publicity |  |  |
| Displacement/gentrification |  |  |
| Poverty |  |  |
| **Aspirations: Community Resources/Quality of Neighborhood Life** | 7.61 | 0.34 |
| Safe walking and biking areas (trails, streets, parks) |  |  |
| Gathering places or spaces (gym, swimming, meeting halls) |  |  |
| Leisure activities |  |  |
| Community garden (education, engagement, youth interaction) |  |  |
| Community volunteering opportunities (available but not frequent enough) |  |  |
| **Aesthetic** | 7.07 | 0.83 |
| Aesthetic (pollinators, holistic) |  |  |
| **Issues: Community Resources/Quality of Neighborhood Life** | 6.90 | 0.44 |
| Lack of interaction between Spelman and West End community |  |  |
| Loitering |  |  |
| Not enough resources to serve community |  |  |
| Neighborhood safety (gun fire) |  |  |
| Security/Safety |  |  |
| Horrible roads everywhere |  |  |
| Outdoor spaces are uninviting (nowhere to walk, safety issues, not enough infrastructure) |  |  |
| Alleys |  |  |
| Streets not made for walking (no sidewalks) |  |  |
| **Development and Growing Pains** | 6.74 | 0.59 |
| Proposed densely populated housing developments (more cars and traffic) |  |  |
| Excessive noise (music, cars, guns, trains) |  |  |
| Airplane overhead always |  |  |
| Transportation |  |  |
| Green public transportation |  |  |

| **Community D: Cluster and Statement List** | | | | | |
| --- | --- | --- | --- | --- | --- |
| **Cluster Names and Statements** | **Average Cluster Rating**  **(1-10)** | | | **Average Bridging**  **Value** | |
| **Public Safety** | 9.03 | | | 0.98 | |
| Crime |  | | |  | |
| Safety |  | | |  | |
| **Environmental Issues** | 8.94 | | | 0.29 | |
| Unknown environmental issues |  | | |  | |
| Water pollution |  | | |  | |
| Toxic waste going through the community |  | | |  | |
| Air pollution/quality |  | | |  | |
| Safe drinking water |  | | |  | |
| Soil issues |  | | |  | |
| Birds of prey |  | | |  | |
| South Dekalb land fill |  | | |  | |
| **Government issues** | 8.13 | | | 0.72 | |
| Lack of responsiveness from local government |  | | |  | |
| Lack of transparency |  | | |  | |
| Traffic |  | | |  | |
| **Community Initiatives** (5) | 8.02 | | | 0.6 | |
| Lack of park space in community |  | | |  | |
| Types of businesses in community | | |  |  | |
| Change/growth | | |  |  | |
| Beautification of community | | |  |  | |
| Rezoning issues | | |  |  | |
